# Supplementary figures and images for: Quantitative ethology of schistosome miracidia characterizes a conserved snail peptide that inhibits penetration
Source: bioRxiv. 2025 Jul 3:2025.07.02.662618. Preprint. [Version 1] doi: 10.1101/2025.07.02.662618 (PMC12236672; doi:10.1101/2025.07.02.662618)

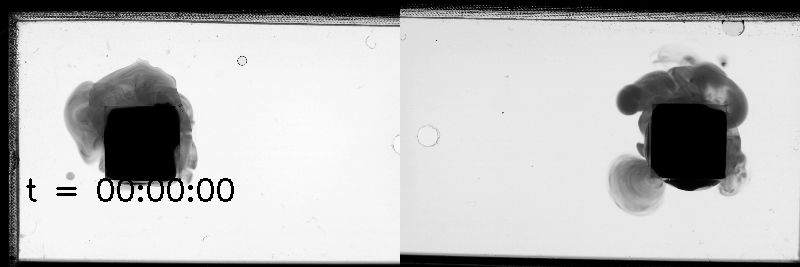

Supplement: Supplement 1 [file media-1.gif]

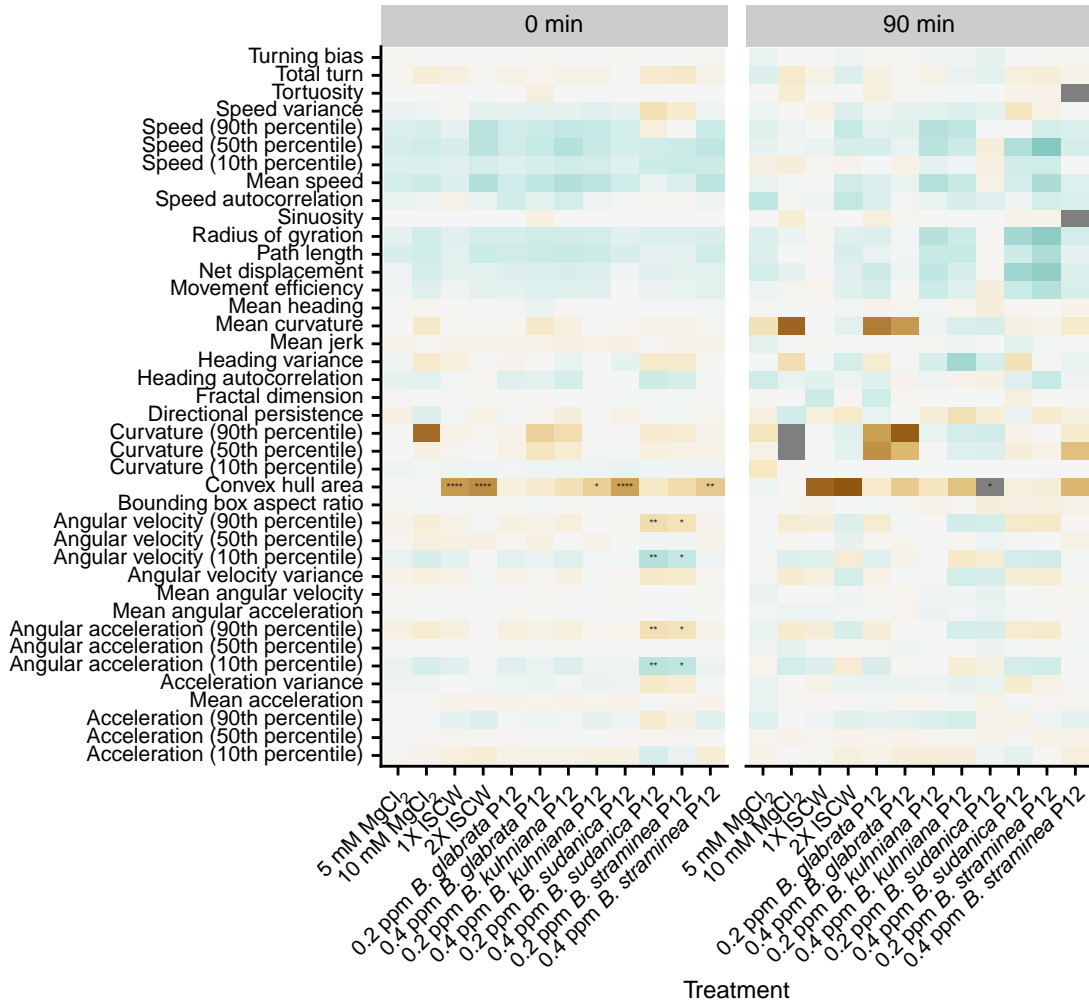

Standardized  
effect size

-1.0 -0.5 0.0 0.5 1.0

Supplement: Supplement 2 [file media-2.pdf]

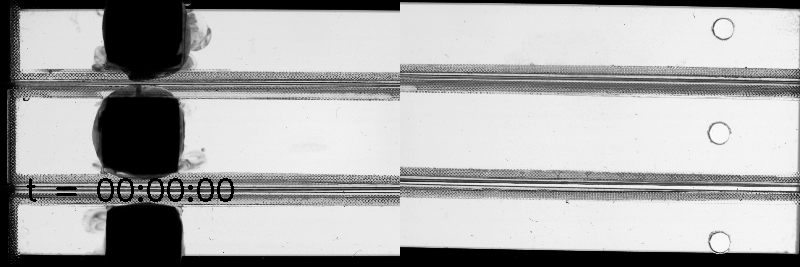

Supplement: Supplement 3 [file media-3.gif]
